# Venoarterial Extracorporeal Membrane Oxygenation in High-Risk Pulmonary Embolism: A Case Series and Literature Review

**DOI:** 10.31083/j.rcm2306193

**Published:** 2022-05-27

**Authors:** Zied Ltaief, Ermes Lupieri, Jean Bonnemain, Nawfel Ben-Hamouda, Valentina Rancati, Sabine Schmidt Kobbe, Matthias Kirsch, Jean-Daniel Chiche, Lucas Liaudet

**Affiliations:** ^1^Service of Adult Intensive Care Medicine, Lausanne University Hospital, 1010 Lausanne, Switzerland; ^2^Service of Anesthesiology, Lausanne University Hospital, 1010 Lausanne, Switzerland; ^3^Service of Radiology, Lausanne University Hospital, 1010 Lausanne, Switzerland; ^4^Service of Cardiac Surgery, Lausanne University Hospital, 1010 Lausanne, Switzerland

**Keywords:** pulmonary embolism, cardiac arrest, obstructive cardiogenic shock, cardiopulmonary resuscitation, extra-corporeal cardiopulmonary resuscitation (ECPR), veno-arterial extra-corporeal membrane oxygenation (VA-ECMO)

## Abstract

**Background:**

High-risk Pulmonary Embolism (PE) has an ominous prognosis and requires emergent reperfusion therapy, primarily systemic thrombolysis (ST). In deteriorating patients or with contraindications to ST, Veno-Arterial Extracorporeal Membrane Oxygenation (VA-ECMO) may be life-saving, as supported by several retrospective studies. However, due to the heterogeneous clinical presentation (refractory shock, resuscitated cardiac arrest (CA) or refractory CA), the real impact of VA-ECMO in high-risk PE remains to be fully determined. In this study, we present our centre experience with VA-ECMO for high-risk PE.

**Method:**

From 2008 to 2020, we analyzed all consecutive patients treated with VA-ECMO for high-risk PE in our tertiary 35-bed intensive care unit (ICU). Demographic variables, types of reperfusion therapies, indications for VA-ECMO (refractory shock or refractory CA requiring extra-corporeal cardiopulmonary resuscitation, ECPR), hemodynamic variables, initial arterial blood lactate and ICU complications were recorded. The primary outcome was ICU survival, and secondary outcome was hospital survival.

**Results:**

Our cohort included 18 patients (9F/9M, median age 57 years old). VA-ECMO was indicated for refractory shock in 7 patients (2 primary and 5 following resuscitated CA) and for refractory CA in 11 patients. Eight patients received anticoagulation only, 9 received ST, and 4 underwent surgical embolectomy. ICU survival was 1/11 (9%) for ECPR vs 3/7 (42%) in patients with refractory shock (*p* = 0.03, log-rank test). Hospital survival was 0/11 (0%) for ECPR vs 3/7 for refractory shock (*p* = 0.01, log-rank test). Survivors and Non-survivors had comparable demographic and hemodynamic variables, pulmonary obstruction index, and amounts of administered vasoactive drugs. Pre-ECMO lactate was significantly higher in non-survivors. Massive bleeding was the most frequent complication in survivors and non-survivors, and was the direct cause of death in 3 patients, all treated with ST.

**Conclusions:**

VA-ECMO for high-risk PE has very different outcomes depending on the clinical context. Furthermore, VA-ECMO was associated with significant bleeding complications, with more severe consequences following systemic thrombolysis. Future studies on VA-ECMO for high-risk PE should therefore take into account the distinct clinical presentations and should determine the best strategy for reperfusion in such circumstances.

## 1. Introduction

High-risk pulmonary embolism (PE) is defined as acute PE with hemodynamic instability, characterized either as persistent hypotension (systolic blood pressure (SBP) less than 90 mmHg for more than 15 min without signs of organ hypoperfusion), obstructive shock (—SBP less than 90 mmHg with signs of organ hypoperfusion), or cardiac arrest (CA) [1]. While only 4 to 5% of PE are considered high-risk, they account for most PE-related early death [2, 3], with reported mortality rates up to 95% in patients presenting with cardiac arrest [4].

Recently updated guidelines recommend emergent reperfusion therapy for high risk PE, primarily with systemic thrombolysis (class I, level of evidence B), or with surgical embolectomy or catheter-directed therapy (CDT) in case of contraindications to systemic thrombolysis [1]. The use of veno-arterial extracorporeal membrane oxygenation (VA-ECMO) has also been advocated as a life sustaining therapy for patients presenting with refractory shock or cardiac arrest in the setting of high-risk PE [5], while awaiting the resolution of pulmonary artery obstruction. Therefore, and despite the lack of solid level evidence studies, European society of cardiology guidelines indicate that VA-ECMO may be considered (Class IIb, level C) in patients with intractable circulatory collapse related to PE [1].

Due to the current absence of prospective randomized study evaluating VA-ECMO in high risk PE, its potential benefits in this setting have only been presented in case reports and retrospective case series. Although most of these studies reported survival benefits from VA-ECMO, interpretation of these results is hampered by important limitations. The first one is the lack of formal diagnosis of PE as a cause of CA in a proportion of reported cases, where the observation of an acutely dilated right ventricle (RV) was considered as indirect evidence of acute PE. However, the RV may also acutely dilate during CA caused by arrhythmias, hyperkalemia and hypovolemia, hence independently from pulmonary obstruction [6]. The second limitation refers to the indication of VA-ECMO to provide circulatory support in cardiac arrest due to PE. VA-ECMO may indeed provide mechanical support for refractory shock following the return of spontaneous circulation (ROSC) after conventional cardiopulmonary resuscitation (CPR), or may be used for extracorporeal cardiopulmonary resuscitation (ECPR) in refractory cardiac arrest [7]. In the latter, the chances of survival are expected to be low, due to the absence of transpulmonary blood flow during CPR prior to the insertion of ECMO. Results of VA-ECMO for PE-associated cardiac arrest should therefore take into account this important distinction.

In this study, we present a retrospective case series of 18 patients treated in our tertiary-care centre with VA-ECMO for high-risk PE. We report the outcomes, associated therapies and complications, according to the indications of VA-ECMO in this population (ECPR for refractory CA or mechanical support for refractory shock).

## 2. Materials and Methods

### 2.1 Study Setting

**Fig. 1.**
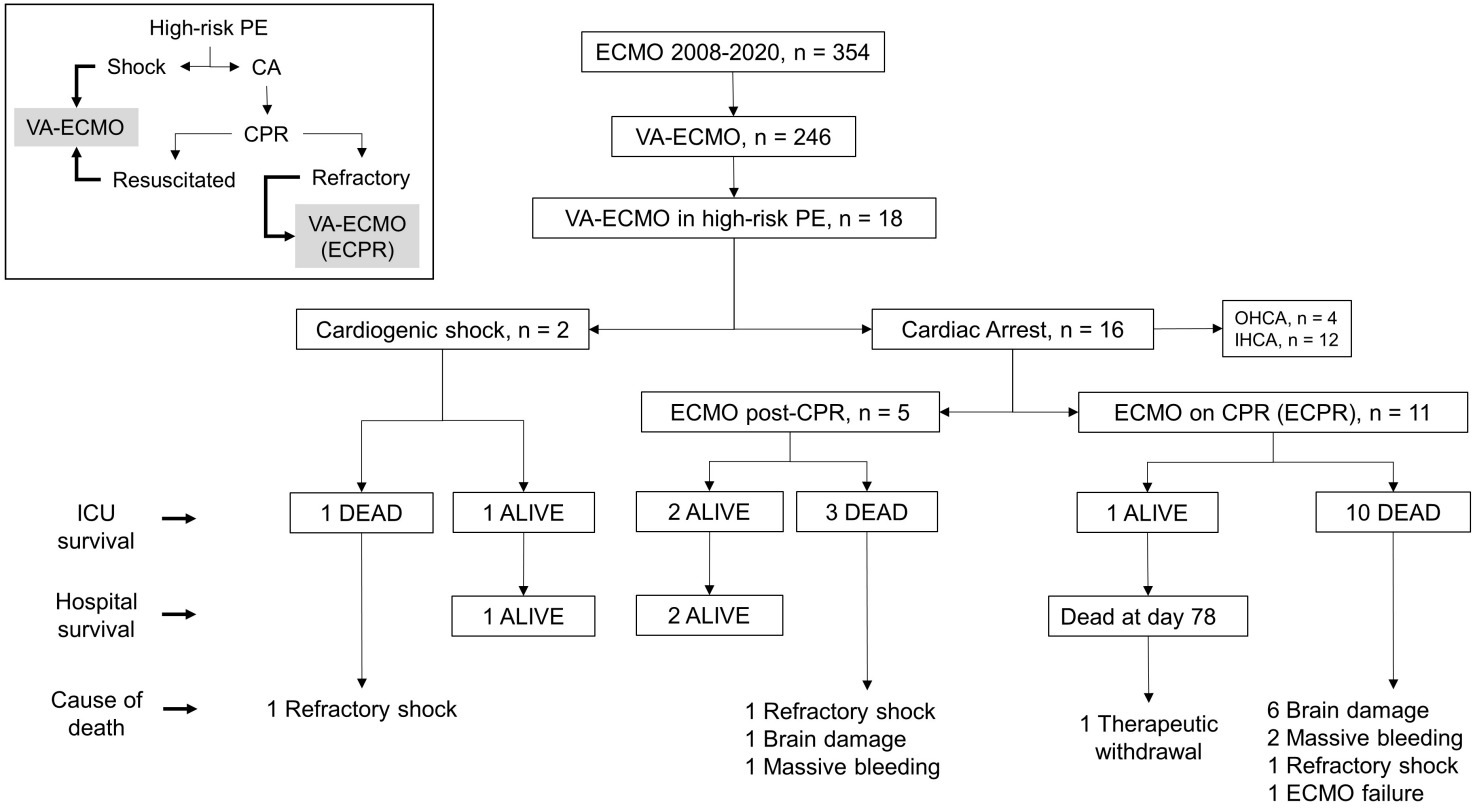
**Study flow chart**. The insert on the top left indicates the different clinical presentations of high-risk PE in which VA-ECMO was indicated. CPR, Cardiopulmonary Resuscitation; ECPR, Extracorporeal Cardiopulmonary Resuscitation; PE, Pulmonary Embolism; VA-ECMO, Veno-Arterial Extracorporeal Membrane Oxygenation; CA, cardiac arrest; ICU, intensive care unit; OHCA, Out-of-Hospital CA; IHCA, In-Hospital CA.

This retrospective study was approved by our local ethical committee with waiver of consent (Commission Cantonale d’Ethique de la Recherche sur l’Etre Humain/CER-VD-Nr: 2017–01184). The cohort included 18 patients treated with VA-ECMO for high-risk PE in our 35-bed multidisciplinary ICU from 2008 to 2020, who were included in our local database of 246 patients treated with VA-ECMO during this period, as indicated in the flow chart of the study (Fig. 1). Our study was strictly limited to high-risk PE patients undergoing VA-ECMO, and we did not include a cohort patients with high-risk PE not treated with VA-ECMO. Our study conforms with the STROBE guidelines for the reporting of retrospective studies.

### 2.2 VA-ECMO Treatment

In the absence of a specific protocol for VA-ECMO in high risk PE, the decision to start VA-ECMO was taken by the physicians in charge of the patient on admission, in the presence of: (1) Refractory shock, defined by hypotension requiring high dose catecholamines and ongoing tissue hypoxia (lactic acidosis). (2) Refractory CA (absence of ROSC after at least 20 min CPR in patients with a no flow time <5 min [8]). The insertion of VA-ECMO was performed by cardiac surgeons, primarily via a femoro-femoral approach. In a subset of patients, ECMO was surgically inserted via central cannulation (see results). Initial VA-ECMO settings targeted a blood flow of 40–60 mL/kg, with a sweep fraction of oxygen (FSO2) set at 100%, and gas flow adapted to maintain normal PaCO2. Vasopressors and inotropes (Noradrenaline, Adrenaline, Dobutamine), as well as intravenous (IV) fluids were given to maintain the target blood flow and mean blood pressure (BP) ≥65 mmHg. Patients were mechanically ventilated at an FiO2 initially set at 100%, a tidal volume of 6–8 mL/kg, a respiratory rate of 10–20/min and a positive end expiratory pressure (PEEP) of 5 cm H2O. Sedation was maintained with Midazolam (0.05–0.15 mg/h) or Propofol (2–4 mg/kg/h). ECMO was discontinued in the presence of irreversible circulatory shock, intractable massive haemorrhage or evidence of severe neurological injury (major brain damage on computed tomography (CT)-scan or evidence of severe anoxic brain injury as determined by multimodal outcome prediction) [8, 9]. Criteria for ECMO weaning included a mean blood pressure >65 mmHg and echocardiographic evidence of cardiac recovery, with an aortic velocity time integral >10 cm/sec, under minimal vasopressor and inotropic support [8].

### 2.3 Data Collection

Demographic variables included age, sex, body mass index, the prevalence of co-morbidities and Apache II score. We recorded the number of patients with formally documented PE and the number of patients experiencing CA, for which we determined the location (OHCA: Out-of-Hospital CA; IHCA: In-Hospital CA), initial rhythm, the duration of no flow and low flow, as well as the type of resuscitation (conventional CPR or ECPR).

Hemodynamic variables included mean BP, heart rate, arterial blood pH and lactate on admission. In patients undergoing thoracic CT scan, we calculated the pulmonary obstruction index and the ratio of right ventricle to left ventricle diameter.

Treatment data included the modality of VA-ECMO (peripheral versus central cannulation), the administration of systemic thrombolytic agents, catheter-directed therapy (CDT), surgical embolectomy and anticoagulation, the amount of intravenous fluids, vasopressors and inotropes administered during the first 24 h, as well as the vasoactive-inotropic score, calculated for the first 24 h [5, 10]. We also collected the amount of packed red blood cells, platelets and fresh frozen plasma administered, and the proportion of patients requiring renal replacement therapy.

Outcome variables included ICU and in-hospital mortality. We also determined the causes of death, ICU and hospital length of stay, the duration of ECMO treatment and ICU complications.

### 2.4 Data Analysis

Continuous variables are expressed as medians and interquartile range (IQR), and categorical data are presented as numbers and percentages. The primary outcome was ICU mortality and the secondary outcome was survival to hospital discharge. Mortality was assessed using Kaplan–Meier curves, and any differences were investigated through the log-rank test. All other comparison between survivors and non-survivors were done using the Student’s *t* test or the Mann-Whitney test when appropriate for continuous variables, and the chi-square tests for categorical data. A *p*-value < 0.05 was considered statistically significant. Statistical analyses were performed using the JMP software, version 15 (Copyright © SAS Institute Inc., SAS Campus Drive, Cary, North Carolina, USA).

## 3. Results

### 3.1 Demographic Data, Clinical Characteristics, Diagnosis and Management of PE, and Outcome of VA-ECMO

Demographic data and clinical characteristics are shown in Table 1, and the detailed presentation of the patients is given in Table 2. The cohort included 18 patients (M/F 9/9, median age 57). ICU Survivors (n = 4) and non-survivors (n = 14) displayed statistically comparable Apache II score, body mass index (BMI) and co-morbidities. The ICU and hospital length of stay (LOS), as well as the duration of mechanical ventilation were all statistically significantly longer in survivors vs non-survivors.

**Table 1.** **Demographic variables, diagnosis and management of PE**.

Variable		All (n = 18)	Alive (n = 4)	Dead (n = 14)
Age, yr		57 (47–66)	52 (40–57)	67 (54–72)
Male, n (%)		9 (50)	2 (50)	7 (50)
Apache II, median (IQR)		35 (31–43)	35 (31–41)	42 (16–44)
BMI, kg/$m^{2}$, median (IQR)		30 (23–42)	30 (23–34)	27 (22–30)
Hypertension, n (%)		7 (38)	2 (50)	5 (35)
Diabetes, n (%)		2 (11)	1 (25)	1 (7)
Chronic heart disease, n (%)		6 (33)	1 (25)	5 (35)
COPD, n (%)		1(5)	1 (25)	0 (0)
ICU LOS, days, median (IQR)		1.6 (0.8–8.3)	19.6 (7.1–55.2)	1.2 (0.7–3.0) *
Hospital LOS, days, median (IQR)		2.9 (1.0–9.9)	37.0 (7.1–74.5)	1.6 (0.8–5.4) *
MV duration, hours, median (IQR)		31 (16–57)	249 (116–988)	26 (15–52) *
PE diagnosis				
	CT Scan, n (%)	13 (72)	4 (100)	9 (64)
	Echocardiography, n (%)	1 (5)	0 (0)	1 (7)
	Autopsy, n (%)	2 (15)	0 (0)	2 (14)
	RV dilation + DVT, n (%)	2 (12)	0 (0)	2 (14)
PE specific management				
	Anticoagulation only, n (%)	8 (44)	1 (25)	7 (50)
	Systemic thrombolysis, n (%)	9 (50)	3 (75)	6 (42)
	CDT, n (%)	1 (5)	1 (25)	0 (0) *
	Surgical embolectomy, n (%)	4 (22)	1 (25)	3 (29)

BMI, Body Mass Index; CDT, Catheter-Directed Therapy; COPD, Chronic Pulmonary Obstructive Disease; DVT, Deep Vein Thrombosis; LOS, Length Of Stay; MV, Mechanical Ventilation; RV, Right Ventricle; ICU, Intensive Care Unit; PE, Pulmonary Embolism; CT, Computed Tomography; IQR, Interquartile Range. * *p *< 0.05 Survivors vs Non-Survivors.

**Table 2.** **Characteristics of the patient population**.

Patient	Age	Sex	Underlying cause	Management	CA	LF	ECPR	Survival	Cause of death
P1	66	F	Thrombophilia, DVT	AC	YES	87	YES	NO	Refractory Shock
P2	49	M	Orthopedic surgery	AC	YES	60	YES	NO	Brain Damage
P3	51	F	-	Lysis	YES	65	YES	NO	Brain Damage
P4	32	F	Heart Failure, DVT	AC	NO	-	-	NO	Refractory shock
P5	64	F	DVT	Lysis	YES	20	NO	YES	-
P6	51	M	Cancer, DVT	Lysis, CDT, SE	NO	-	-	YES	-
P7	73	M	DVT	AC, SE	YES	50	YES	YES	*
P8	64	M	Cancer, Abdominal surgery	Lysis, SE	YES	30	NO	NO	Refractory Shock
P9	48	F	Cardiac Surgery, HIT, DVT	SE	YES	5	YES	NO	ECMO failure, HIT
P10	71	F	Abdominal surgery	Lysis	YES	20	YES	NO	Massive Bleeding
P11	67	F	Orthopedic surgery, DVT	Lysis, SE	YES	20	NO	NO	Brain Damage
P12	20	M	Orthopedic surgery	Lysis	YES	45	YES	NO	Massive Bleeding
P13	53	M	-	Lysis	YES	15	NO	NO	Massive Bleeding
P14	71	F	DVT	Lysis	YES	15	NO	YES	-
P15	43	M	-	AC	YES	45	YES	NO	Brain Damage
P16	66	M	Orthopedic surgery, DVT	AC	YES	90	YES	NO	Brain Damage
P17	62	M	Prostate Cancer, Surgery	AC	YES	50	YES	NO	Brain Damage
P18	23	F	DVT	AC	YES	70	YES	NO	Brain Damage

AC, Anticoagulation; CA, Cardiac Arrest; CDT, Catheter-Directed Therapy; DVT, Deep Vein Thrombosis; ECPR, Extracorporeal Cardiopulmonary Resuscitation; HIT, Heparin-Induced Thrombocytopenia; LF, Low Flow; PE, Pulmonary Embolism; SE, Surgical Embolectomy; ECMO, Extracorporeal Membrane Oxygenation. * Patient 7 died at day 78 from complications of mesenteric ischemia (therapeutic withdrawal). In patient 9, CA occurred in the operating room at the onset of surgical embolectomy, resulting in a low flow time of only 5 minutes.

The diagnosis of PE was formally documented on imaging (n = 14) or autopsy (n = 2) in 16 patients (89%). In two patients, PE was considered highly probable, owing to the presence of severe right heart dysfunction on echography and evidence of deep vein thrombus in the post-operative period (day 3 post-orthopedic surgery in 1 patient, day 6 post-laparotomy, in 1 patient). Specific therapy for PE included anticoagulation only in 8 patients, systemic thrombolysis in 9 patients, and surgical embolectomy in 4 patients, with no difference between survivors and non survivors. Catheter-directed therapy was performed in one patient who ultimately survived. Four patients (2 survivors and 2 non-survivors) received more than one specific therapy for PE.

**Fig. 2.**
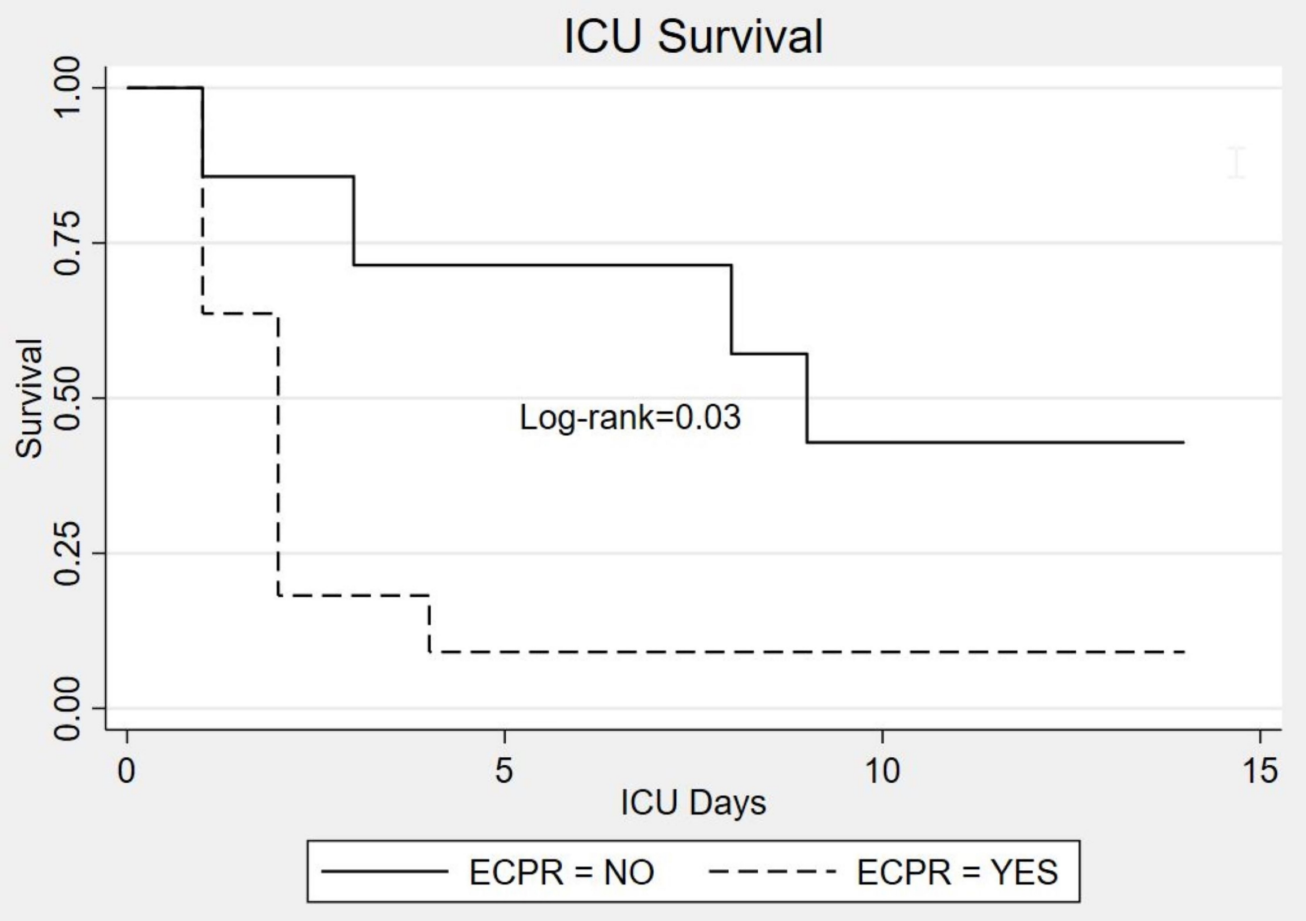
**Kaplan-Meier probability of ICU survival**. ECPR, Extracorporeal Cardiopulmonary Resuscitation; ICU, Intensive Care Unit.

Kaplan-Meyer curves of ICU survival is depicted in Fig. 2. Overall ICU survival was 22% (4/18 patients). According to ECMO indications, survival was 9% (1/11 patients) for ECPR and 42% (3/7 patients) for VA-ECMO in refractory shock (*p* = 0.03, log-rank test). The causes of deaths, as indicated in Fig. 1, were anoxic encephalopathy (n = 7), refractory shock (n = 3), intractable bleeding (n = 3) and acute ECMO membrane dysfunction due to heparin-induced thrombocytopenia in 1 patient. Hospital survival (secondary outcome) was 0% (0/11 patients) for ECPR and 42% (3/7 patients) for VA-ECMO in refractory shock (*p* = 0.01, log-rank test). The only ICU survivor of ECPR died at hospital day 78 from complications related to mesenteric ischemia, leading to therapeutic withdrawal.

### 3.2 Characteristics of CA in the Study Population

A majority of patients in our cohort suffered pre-ECMO CA (16/18 = 89%), including 4 OHCA and 12 IHCA (Table 3). All but one patients presented with an initial non-shockable rhythm. For all CA, no flow time was <2 min and median low flow time was 45 min, with a non-significant trend towards shorter low flow time in survivors (median 20 min) than non-survivors (median 45 min). Five patients disclosed ROSC during conventional CPR. In these patients, VA-ECMO was inserted for refractory shock post-ROSC. Three of them died and two survived. Eleven patients underwent ECPR, with VA-ECMO being inserted during CPR for refractory CA. The median low flow time was 50 min, and ICU survival was observed only in one patient.

**Table 3.** **Characteristics of cardiac arrest in the study population**.

All cardiac arrest		All CA (n = 16)	Alive (n = 3)	Dead (n = 13)
	OHCA, n (%)	4 (25)	1 (33)	3 (23)
	IHCA, n (%)	12 (75)	2 (66)	10 (76)
	Shockable rhythm, n (%)	1 (6)	0 (0)	1 (7)
	No Flow <2 mn, n (%)	16 (100)	3 (100)	13 (100)
	Low Flow, min (median, IQR)	45 (20–62)	20 (15–50)	45 (20–65)
Resuscitated CA		All (n = 5)	Alive (n = 2)	Dead (n = 3)
	OHCA, n (%)	1 (20)	1 (50)	0 (0)
	IHCA, n (%)	4 (80)	1 (50)	3 (100)
	Shockable rhythm, n (%)	0 (0)	0 (0)	0 (0)
	No Flow <2 mn, n (%)	5 (100)	2 (100)	3 (100)
	Low Flow, min (median, IQR)	20 (15–20)	17 (15–20)	20 (15–30)
Refractory CA		All (n = 11)	Alive (n = 1)	Dead (n = 10)
	OHCA, n (%)	3 (27)	0 (0)	3 (30)
	IHCA, n (%)	8 (72)	1 (100)	7 (70)
	Shockable rhythm, n (%)	1 (9)	0 (0)	1 (10)
	No Flow <2 mn, n (%)	11 (100)	1 (100)	10 (100)
	Low Flow, min (median, IQR)	50 (45–70)	50	55 (45–70)

All *p* values > 0.05. CA, Cardiac Arrest; OHCA, Out-Of-Hospital Cardiac Arrest; IHCA, In-Hospital Cardiac Arrest.

### 3.3 VA-ECMO Characteristics and Complications

As shown in Table 4, VA-ECMO was implanted peripherally in 14 patients, and 4 patients received central ECMO at the time of surgical embolectomy (*p* = NS, survivors vs non-survivors). The indications of ECMO included ECPR for refractory CA in 11 patients or mechanical circulatory support for refractory shock in 7 patients. All ECPR were performed with peripheral ECMO implantation, except in 1 patient who experienced CA at the time of surgical embolectomy, leading to central cannulation for ECPR.

**Table 4.** **VA-ECMO characteristics, complications and outcomes**.

VA-ECMO characteristics	All Patient n = 18	Survivors n = 4	Non Survivors n = 14
Central ECMO, n (%)	4 (22)	1 (25)	3 (21)
Peripheral ECMO, n (%)	14 (78)	3 (75)	11 (78)
VA-ECMO for refractory shock, n (%)	7 (39)	3 (75)	4 (28) ^#^
Post-CA cardiogenic shock, n (%)	5 (28)	2 (50)	3 (21)
Primary cardiogenic shock, n (%)	2 (11)	1 (25)	1 (7)
ECPR for refractory CA, n (%)	11 (61)	1 (25)	10 (71) ^#^
ECMO duration, hours (median, IQR)	30 (14.5–70.8)	76.5 (40–124.3)	26.5 (11.5–40.3) *
ECMO weaning, n (%)	6 (33)	4 (100)	2 (14) *
VA-ECMO complications			
Brain Damage, n (%) ^a^	7 (50)	0 (0)	7 (50) ^§^
Infection, n (%) ^b^	4 (22)	2 (50)	2 (14)
Mesenteric ischemia	2 (11)	1 (25)	1 (7)
RRT, n (%)	8 (44)	3 (75)	5 (35)
Massive Bleeding, n (%)	13 (72)	3 (75)	10 (71)
Packed red-cell, units (median, IQR)	5.0 (2.0–12.8)	11.5 (3.5–18.0)	3 (2.0–10.5)
FFP, units (median, IQR)	2.5 (0.0–10.3)	6.0 (3.0–18.0)	1.5 (0.0–10.3)
Platelets, units (median, IQR)	0.0 (0.0–1.5)	2.0 (0.3–5.3)	0 (0.0–0.0) *

^a^ Brain damage: Anoxic encephalopathy (4); Massive brain edema on CT scan (3); ^b^ Infection: Mediastinitis (1); Pneumonia (2); Septic shock (1). CA, Cardiac Arrest; VA-ECMO, Veno-Arterial Extracorporeal Membrane Oxygenation; ECPR, Extracorporeal Cardio-Pulmonary Resuscitation; FFP, Fresh-Frozen Plasma; RRT, Renal Replacement Therapy. * *p *< 0.05; # *p* = 0.09; §* p* = 0.07.

Complications related to VA-ECMO included anoxic encephalopathy in 7 patients (massive brain edema on CT scan in 2 patients, areactive electroencephalogram (EEG) and absent somesthesic evoked potential in 5 patients), infection in 4 patients, mesenteric ischemia in 2 patients and acute renal failure in 8 patients. Massive bleeding, as defined according to the ISTH classification (International Society of the Thrombosis and Hemostasis) [11] occurred in 13 patients, with no significant difference between survivors (3/4 patients 75%) and non-survivors (10/14 patients, 71%). Bleeding was the direct cause of death in 3 patients. Death from massive bleeding was significantly more common in patients receiving systemic thrombolysis (*p* = 0.05).

### 3.4 Hemodynamic Data and Management 

As indicated in Table 5, survivors and non-survivors had comparable values of mean arterial pressure and arterial pH at admission. Non-survivors disclosed a slower heart rate (*p* = 0.07 vs survivors) and higher arterial blood lactate. The pulmonary obstruction index tended to be higher in non-survivors, but the difference with survivors was not significant. The Right-to-Left ventricular ratio was comparable in survivors and non-survivors, as were the doses of catecholamines (Norepinephrine, Dobutamine and Epinephrine), the vasoactive-inotropic score and the amount of administered fluids during the first 24 h.

**Table 5.** **Hemodynamic data on admission and treatments**.

Variable	All Patient n = 18	Alive (n = 4)	Dead (n = 14)
MAP, mmHg	76 (66–88)	75 (66–93)	76 (63–88)
HR, bpm	96 (74–115)	124 (89–135)	88 (66–106) ^#^
Lactate, mmol/L	12.3 (8.9–20.0)	9.0 (2.6–12.5)	12.4 (9.6–21.0) *
pHa	7.06 (6.88–7.30)	7.15 (6.67–7.47)	7.06 (6.88–7.24)
Pulmonary obstruction index	35 (25–69)	30 (24–55)	48 (25–75)
Right to Left Ventricular ratio	1.7 (1.0–2.3)	1.8 (1.0–2.4)	1.7 (1.0–2.3)
Norepinephrine (μg/kg/min)	0.33 (0.17–0.84)	0.40 (0.08–1.06)	0.33 (0.17–0.84)
Epinephrine (μg/kg/min)	0.05 (0.02–0.23)	0.03 (0.01–0.07)	0.05 (0.02–0.28)
Dobutamine (μg/kg/min)	0.00 (0.00 –0.17)	0.03 (0.00–2.36)	0.00 (0.00–0.17)
Vasoactive-inotropic score	63.6 (31.0–112.2)	48.7 (33.9–135.3)	66.1 (10.3–110.8)
Fluids (mL/kg/h)	4.6 (3.7–7.4)	4.7 (3.5–7.7)	4.6 (3.8–7.7)

MAP, mean arterial pressure; HR, heart rate. Categorical variables are expressed as n (%) and continuous variables as median and IQR (25–75). Data on therapies indicate values for the first 24 h. * *p *< 0.05; ^#^*p* = 0.07.

## 4. Discussion

The main results of our study are that VA-ECMO in high-risk PE was associated with a ICU survival of 22%, with only 9% survivors when VA-ECMO was inserted during CPR for refractory CA (ECPR), contrasting with 42% survival in patients treated with VA-ECMO for refractory shock, either primary or following resuscitated CA.

High-risk PE (massive PE), characterized by profound hemodynamic instability, is associated with a particularly high mortality, ranging from 47 to 52% in the absence of CA [3, 12] to 84 to 95% when PE is complicated by CA [4, 12]. This ominous prognosis reflects the inability to maintain systemic perfusion due to the obstruction of the pulmonary arteries (PA), leading to acute right ventricle (RV) overload [5]. Hence, treatment of high-risk PE must include a strategy to remove pulmonary obstruction, primarily with systemic thrombolysis (grade I recommendation), or, alternatively, with catheter-directed therapy or surgical embolectomy (grade IIa) [1]. In the presence of contraindications to thrombolysis, or in patients presenting with refractory cardiac arrest or deteriorating in spite thrombolysis, mechanical circulatory support with VA-ECMO may represent the only viable strategy to provide adequate systemic perfusion while awaiting the resolution of pulmonary obstruction (bridge-to-therapy or bridge-to-recovery) [13].

The role of VA-ECMO in high-risk PE has been the matter of several retrospective studies, case reports, reviews and meta-analyses. We reviewed 33 publications presenting retrospective analyses of a total of 2996 high-risk PE patients treated with VA-ECMO, as summarized in Tables 6.1,6.2,6.3 (Ref. [5, 7, 14, 15, 16, 17, 18, 19, 20, 21, 22, 23, 24, 25, 26, 27, 28, 29, 30, 31, 32, 33, 34, 35, 36, 37, 38, 39, 40, 41, 42, 43, 44]). Except from one study totalizing 2197 patients, most studies included a limited number of patients (5–87, average 23 patients), and data regarding the specific indication of ECPR were available in 150 patients from 19 studies. We extracted the proportion of patients with a confirmed diagnosis of PE, the proportion of patients with cardiogenic shock and cardiac arrest, as well as the survival rate in each study. The mean overall survival rate reported was 56%, albeit with considerable variability across studies (20–95% reported survival rates). The 22% ICU survival in our cohort appears therefore much less favourable, but several hypotheses may be advanced to explain such high mortality. The first one is related to the high proportion (89%) of patients experiencing CA in our study, and most significantly, to the high percentage of ECPR (61%, with an ICU survival of 9%, contrasting with 42% survival for non-ECPR indications). It is particularly noteworthy that VA-ECMO in high-risk PE encompasses different indications, with distinct pathophysiology and most probably very different outcomes, which comprise refractory shock, resuscitated CA, and refractory CA [14, 15]. Many published studies did not consider this heterogeneity and did not provide details on the relative mortality of VA-ECMO according to these various conditions. This may represent an important drawback for the correct interpretation of the potential benefits of VA-ECMO in high-risk PE, as emphasized by Karami *et al*. [45] in their recent meta-analysis.

**Table 6.1:** **Studies with mortality data on ECPR (classified by decreasing number of patients)**.

n	PE diagnosis	CS	CA	Overall survival	ECPR	ECPR survival	Reference
52	42 (81)	13 (25)	39 (75)	20 (38)	18 (34)	2 (11)	[15]
36	9 (100)	14 (39)	22 (51)	23 (64)	13 (36)	5 (38)	[5]
36	36 (100)	21 (59)	24 (67)	24 (67)	9 (25)	0 (0)	[16]
32	32 (100)	17 (53)	15 (46)	17 (53)	2 (6)	0 (0)	[17]
25	25 (100)	17 (68)	8 (32)	20 (80)	6 (24)	3 (50)	[18]
22	22 (100)	0 (0)	22 (100)	12 (54)	5 (22)	3 (60)	[19]
22	NA	0 (0)	22 (100)	5 (26)	22 (100)	5 (26)	[20]
21	16 (76)	13 (61)	8 (38)	13 (64)	8 (38)	5 (62)	[21]
21	19 (90)	10 (47)	11 (52)	11 (52)	7 (33)	2 (9)	[22]
20	20 (100)	15 (75)	5 (25)	19 (95)	2 (10)	1 (50)	[23]
17	12 (70)	2 (11)	15 (88)	8 (47)	7 (41)	1 (14)	[14]
17	10 (58)	7 (41)	10 (58)	13 (76)	6 (35)	2 (33)	[24]
16	16 (100)	4 (25)	12 (75)	9 (57)	12 (75)	6 (50)	[25]
12	12 (100)	0 (0)	12 (100)	5 (41)	9 (75)	2 (22)	[26]
10	10 (100)	1 (10)	9 (90)	7 (70)	9 (90)	6 (67)	[27]
7	7 (100)	2 (28)	5 (71)	4 (57)	5 (71)	2 (40)	[28]
5	5 (100)	0 (0)	5 (100)	1 (20)	5 (100)	1 (20)	[29]
5	5 (100)	1 (20)	4 (80)	3 (60)	2 (40)	1 (50)	[30]
5	5 (100)	0 (0)	5 (100)	2 (40)	5 (100)	2 (40)	[31]

**Table 6.2:** **Studies without mortality data on ECPR (classified by decreasing number of patients)**.

n	PE diagnosis	CS	CA	Overall survival	ECPR	ECPR survival	Reference
2197	NA	1219 (55)	992 (45)	840 (30)	NA	NA	[7]
87	NA	7 (8)	80 (92)	31 (38)	52 (65)	NA	[32]
83	79 (95)	65 (78)	18 (21)	71 (85)	NA	NA	[33]
75	65 (86)	NA	49 (65)	35 (47)	38 (50)	NA	[34]
29	29 (100)	NA	29 (100)	22 (75)	8 (27)	NA	[35]
27	27 (100)	17 (67)	10 (37)	23 (85)	NA	NA	[36]
18	NA	2 (11)	16 (89)	8 (45)	NA	NA	[37]
18	NA	5 (27)	13 (73)	11 (61)	NA	NA	[38]
17	NA	9 (52)	8 (48)	9 (52)	NA	NA	[39]
14	14 (100)	3 (21)	11 (78)	8 (57)	NA	NA	[40]
13	NA	7 (53)	6 (47)	6 (47)	5 (38)	NA	[41]
13	12 (92)	0 (0)	13 (100)	6 (46)	NA	NA	[42]
12	8 (66)	1 (8)	11 (92)	6 (50)	3 (25)	NA	[43]
12	12 (100)	6 (50)	12 (100)	10 (83)	6 (50)	NA	[44]

**Table 6.3:** **Summary of studies**.

2996 patients	PE diagnosis	CS	CA	Overall survival	ECPR	ECPR survival
N	549	1478	1521	1302	264	49
% (range)	88 (58–100)	56 (0–78)	51 (21–100)	44 (20–95)	43 (6–100)	32 (0–67)

All values are absolute numbers (percentage). In the summary of studies, the percentages have been calculated using as the denominator the number of patients only from studies reporting each given variable. Abbreviations: CA, Cardiac Arrest; CPR, Cardio-Pulmonary Resuscitation; ECPR, Extracorporeal Cardio-Pulmonary Resuscitation; PE, Pulmonary Embolism.

As a matter of fact, it has been clearly shown that the chances of survival are significantly reduced when VA-ECMO is inserted following CA in the setting of PE, most significantly if it is implemented as ECPR for refractory CA [5, 15]. In a large database of 52 patients, Meneveau *et al*. [15] reported an overall survival of 38%, but of only 13% in patients experiencing CA and 11% in those treated with ECPR. Also, Corsi *et al*. [14], showed, in a study on 17 patients, an overall survival of 47%, but 14% in patients treated with ECPR for refractory CA. In one of the largest cohort to date, Giraud *et al*. [5] recently reported that ECMO after or during CA were both independently associated with increased 30 day mortality among PE patients treated with ECMO. Finally, in a systematic review on VA-ECMO in high-risk PE, Scott *et al*. [13] reported a six-fold increase in the risk of death when ECMO was inserted during CPR (ECPR).

These observations emphasize the peculiarities of CA in the context of PE, where obstruction to blood flow in the pulmonary circulation precludes both circulation and gas exchange during CPR. Such phenomenon was well-demonstrated in our patients by a high pulmonary obstruction index (POI), which tended to be greater in non-survivors (47% vs 30%). It is here worth to mention that Van der Meer *et al*. [46] previously reported that a POI greater than 40% was associated with a 11.2-fold increase in mortality in PE patients. In addition, as observed in our study, CA in PE is generally characterized by an initial non-shockable rhythm, known to be correlated with a dismal prognosis in OHCA [47] and IHCA [48].

It is therefore likely that irreversible anoxic end-organ damage may develop during CPR for PE-related CA before the initiation of the artificial circulation, due to poor or absent transpulmonary blood flow. Accordingly, we found that non-survivors displayed a significantly higher value of initial arterial blood lactate (median value 12.4 mmol/L) than survivors, pointing to significant tissue anoxia. In this respect, monitoring the quality of CPR by assessing transpulmonary blood flow using end-tidal CO2 [49] could be particularly useful to determine which patient might or not benefit from VA-ECMO during CPR of PE-related CA, as recently underscored by Giraud *et al*. [5], but such data were unfortunately not available in our patients.

A second hypothesis to explain the higher mortality in our study refers to the high proportion of patients in whom no reperfusion strategy was implemented. Anticoagulation only was administered to 44% of our patients including 50% of non-survivors. This strategy of stand-alone VA-ECMO may have negatively influenced ECMO outcome, as current recommendations suggest that additional therapies to treat embolic obstruction should be considered for best results [1]. In the largest database of ECMO in high-risk PE to date, Hobohm *et al*. [7] showed that the lowest mortality was noted in patients treated with VA-ECMO plus surgical thrombectomy (64%) and VA-ECMO plus thrombolysis (69.7%) in comparison to stand-alone ECMO (72.7%). These results are consistent with those of Meneveau *et al*. [15], who reported a 77.8% mortality of stand-alone VA-ECMO in contrast to only 29.4% of VA-ECMO coupled to surgical embolectomy. These findings support the potential of mechanical embolectomy to improve survival in VA-ECMO for high-risk PE, which should be evaluated in future prospective studies. In our cohort, embolectomy did not appear to show such benefit, with only one surviving patient out of 4 treated with embolectomy.

At variance with the above, Giraud *et al*. [5] reported a significant survival advantage of stand-alone VA-ECMO (85.5% survival) in comparison to VA-ECMO combined with pre-ECMO thrombolysis or CDT (35.5% survival), in a series of 36 VA-ECMO for high-risk PE. As outlined by the authors, the insertion of VA-ECMO after failed thrombolysis (either systemic or catheter-directed) exposes the patient to major risks of severe bleeding, resulting in significant increases in mortality. Indeed, we found a high incidence of hemorrhagic complications in our cohort (72%), occurring at a comparable rate in survivors and non-survivors. Importantly, massive bleeding was the direct cause of death in 3 patients, all of whom had received pre-ECMO systemic thrombolysis. The findings of Giraud *et al*. [5] agree with those of Maggio *et al*. [21], who reported a 77% survival in patients treated with stand-alone VA-ECMO, arguing that in most patients, spontaneous PE lysis generally allows RV recovery within 5 days. Accordingly, Pasrija *et al*. [23] recently proposed a strategy of VA-ECMO with anticoagulation alone, followed by delayed embolectomy (surgical or percutaneous) only in conditions of persistent pulmonary thrombotic obstruction and RV dysfunction.

An obvious development from the above discussion is that adequate patient selection is crucial to optimize VA-ECMO outcome in high-risk PE patients. In the context of refractory shock, VA-ECMO immediately provides adequate systemic perfusion and reduces right ventricle overload, while giving time for spontaneous or mechanical pulmonary reperfusion to restore RV-PA coupling. Early VA-ECMO support and avoidance of thrombolysis to reduce hemorrhagic risk might therefore represent the most effective therapy in such patients. This is indeed supported by the high survival rate reported in studies using such strategy [5, 21, 23]. In contrast, implementing VA-ECMO for refractory CA due to pulmonary obstruction appears futile, due to the considerable risk of irreversible anoxic injury, unless sufficient transpulmonary blood flow can be demonstrated during CPR by the measurement of end-tidal CO2.

An additional factor to consider for the adequate interpretation of survival data relies in the formal diagnosis of PE, documented in 88% of our patients. In previous studies, such documentation has been variable, ranging from 58 to 100% (see Tables 6.1,6.2). Although current recommendations indicate that echocardiographic evidence of acute RV pressure overload in a haemodynamically compromised patients with suspected PE may justify immediate therapy, without the need of confirmatory CT angiography [1], it must be emphasized that RV dysfunction/dilation during or after CA is not specific for acute PE. Aagaard *et al*. [6] demonstrated acute RV dilation in experimental CA (porcine model) induced by hypovolemia, hyperkalemia, and primary arrhythmia. Also, in a retrospective study on 59 patients with CA, Wardi *et al*. [50] found post-CPR RV dysfunction/dilation in the majority of patients, regardless of the aetiology of CA. Accordingly, one may expect that some patients treated with VA-ECMO for suspected PE, solely on the basis of acute RV dysfunction, may have suffered from other causes of CA, with an inherently better prognosis, given the absence of pulmonary vascular obstruction. This might have introduced some bias for the interpretation of survival results in studies without a high level of formal PE confirmation.

Our study has several limitations. First, it was monocentric, retrospective and included a relatively small number of patients, which limited statistical comparisons. It must be noted however that these limitations are shared by many studies in the field, which were all retrospective in design and which reported, with some exceptions, single-center experience of relatively small sample sizes. Obviously, future large-scale prospective studies evaluating VA-ECMO in high-risk PE are warranted to circumvent these limitations. Secondly, we included patients over a 12-year period (2008–2020), with the inherent risk of heterogeneity due to evolving standards of care and practice. Third, the initiation of VA-ECMO in our cohort was decided on a case-by-case basis without a specific protocol, which may have resulted in improper patient’s selection. The implementation of a multidisciplinary pulmonary embolism response team (PERT) [51] might here be helpful to assist the decision making process in the future.

## 5. Conclusions

Our single centre experience of VA-ECMO for documented high-risk PE indicates, in spite of several limitations, that this therapeutic strategy was associated with very different outcomes depending on the clinical context. When used as mechanical circulatory support for refractory shock, whether primary or following resuscitated cardiac arrest, VA-ECMO allowed survival in up to 42% of patients. In contrast, when applied as extracorporeal resuscitation (ECPR) for refractory cardiac arrest, VA-ECMO was associated with only 9% ICU survival. Furthermore, VA-ECMO in high-risk PE was associated with significant bleeding complications, with more severe consequences in patients undergoing systemic thrombolysis. Future studies should therefore take into account the distinct clinical presentations of high-risk PE when reporting the effects of VA-ECMO, and should determine the best strategy for reperfusion therapy in such circumstances.

## Abbreviations

AC, Anticoagulation; BMI, Body Mass Index; CA, cardiac arrest; CDT, Catheter-Directed Therapy; COPD, Chronic Pulmonary Obstructive Disease; CPR, Cardiopulmonary Resuscitation; CS, Cardiogenic Shock; DVT, Deep Vein Thrombosis; ECPR, Extracorporeal Cardiopulmonary Resuscitation; FFP, Fresh-Frozen Plasma; HIT, Heparin-Induced Thrombocytopenia; IHCA, In-Hospital Cardiac Arrest; LOS, Length Of Stay; MV, Mechanical Ventilation; OHCA, Out-Of-Hospital Cardiac Arrest; PE, Pulmonary Embolism; RRT, Renal Replacement Therapy; RV, Right Ventricle; SE, Surgical Embolectomy; VA-ECMO, Veno-Arterial Extracorporeal Membrane Oxygenation; VTE, Venous Thrombo-Embolism.
